# Supplementary material for: Predicting the stalking of celebrities from measures of persistent pursuit and threat directed toward celebrities, sensation seeking and celebrity worship
Source: PLoS One. 2023 Mar 1;18(3):e0281551. doi: 10.1371/journal.pone.0281551 (PMC9977013; doi:10.1371/journal.pone.0281551)
Supplement: S1 File — (DOC) [file pone.0281551.s002.doc]

**Supplementary Material I: OFAS-18**

**Since the age of 16, how often, if at all, have you ever engaged in any of the following activities with a famous celebrity? Use the scale below to record your answer to each item.**

**1 = NEVER 2 = SELDOM 3 = OCCASIONALLY 4 = OFTEN 5 = FREQUENTLY**

**1) TRYING TO MEET THE CELEBRITY FACE-TO-FACE (e.g., going to places you know the person frequents, stand outside their home or workplace, etc,) ____**

**2) OBTAINING PERSONAL ITEMS FROM THE CELEBRITY OR CELEBRITY’S AGENT (e.g., a handkerchief, pen, cap, etc.) ____**

**3) FOLLOWING THE CELEBRITY WHILE OUT IN PUBLIC**

**(e.g., by car, in a store, restaurant, etc.) ____**

**4) GOING TO OR WAITING AT THE CELEBRITY’S HOTEL ____**

**5) TRESPASSING ON THE CELEBRITY’S PROPERTY ____**

**6) EXPRESS ATTRACTION OR SEXUAL INTEREST IN (e.g., statements or offers to engage in romantic or sexual interaction) ____**

**7) ATTEMPTING TO HACK INTO THE CELEBRITY’S PERSONAL OR WORK COMPUTER ____**

**8) SENDING THREAT(S) OR THREATENING OBJECT(S) (e.g., leaving images or objects where the celebrity can find them that imply something bad may happen) ____**

**9) LEAVING OR SENDING UNSOLICITED GIFTS (e.g., flowers, stuffed animals, photographs, jewelry, etc.) ____**

**10) LEAVING OR SENDING MESSAGES OF AFFECTION (e.g., romantically oriented notes, cards, letters, voice mail, twitter, e-mail, messages with friends, etc.) ____**

**11) INVADING HER/HIS PERSONAL SPACE (e.g., getting too close to her/him in conversation, touching her/him, etc.) ____**

**12) INTRUDING UPON HER/HIS FRIENDS, FAMILY, OR COWORKERS (e.g., trying to befriend the celebrity’s friends, family or coworkers; seeking to be invited to social events, seeking employment at their work, etc.) ____**

**13) MONITORING HER/HIM OR HER/HIS BEHAVIOR (e.g., calling at all hours to check on the celebrity’s whereabouts, checking up on the celebrity through mutual friends, etc.) ____**

**14) ENGAGING IN REGULATORY HARRASSMENT (e.g., filing official complaints, spreading false rumors to officials – boss, publicists, tabloids, etc., obtaining a restraining order on her/him, etc.) ____**

**15) STEALING OR DAMAGING VALUED POSESSIONS (e.g., vandalized the celebrity’s property; things taken, damaged or hurt that only the celebrity had access to, such as prior gifts, pets, etc.) ____**

**16) THREATENING OTHERS SHE/HE CARES ABOUT (e.g., threatening harm to or making vague warnings about romantic partners, friends, family, pets, etc.) ____**

**17) GETTING IN TROUBLE OVER ATTEMPTS TO CONTACT THE CELEBRITY (e.g., getting thrown out of an event, being blocked by security persons, etc.) ____**

**18) PHYSICALLY THREATENING THE CELEBRITY (e.g., throwing something at the celebrity, acting as if you will hit the celebrity, running finger across neck implying throat slitting, etc.) ____**

**Supplementary Material II: Skewness and Kurtosis for Each Measure**

|  | Skewness (SD) | Kurtosis (SD) |
| --- | --- | --- |
| Obnoxious Fan AS-18 | 7.13 (0.10) | 59.55 (0.20) |
| CAS Entertainment Social | -0.29 (0.10) | -0.14 (0.20) |
| CAS Intense Personal | 1.06 (0.10) | 0.94 (0.20) |
| CAS Borderline Pathological | 0.62 (0.10) | 0.16 (0.20) |
| ORI & CS Persistent Pursuit | 1.67 (0.10) | 5.35 (0.20) |
| ORI & CS Threat | 3.87 (0.10) | 18.84 (0.20) |
| BSSS Experience Seeking | -0.38 (0.10) | -0.06 (0.20) |
| BSSS Boredom Susceptibility | 0.20 (0.10) | 0.02 (0.20) |
| BSSS Thrill & Adventure Seeking | -0.11 (0.10) | -0.77 (0.20) |
| BSSS Disinhibition | 0.15 (0.10) | -0.50 (0.20) |
| RQ (attachment) | 0.25 (0.10) | -1.95 (0.20) |
| MAI-B (anger) | 0.90 (0.10) | 0.27 (0.20) |
