# Supplementary material for: Predicting the stalking of celebrities from measures of persistent pursuit and threat directed toward celebrities, sensation seeking and celebrity worship
Source: PLoS One. 2023 Mar 1;18(3):e0281551. doi: 10.1371/journal.pone.0281551 (PMC9977013; doi:10.1371/journal.pone.0281551)
Supplement: Attachment — Submitted filename: response to editors & reviewers plos 10-29.docx [file pone.0281551.s003.docx]

October 29, 2022

Dear Dr. Rowe:

My coauthors and I are grateful to you and the two reviewers for the comments and suggestions. Reviewers pointed out strengths of the manuscript and issues that need to be addressed. We responded to their comments and suggestions below. Whenever possible, changes were made in the revised manuscript. When changes were not made, we provided an explanation. Changes were noted in the revised manuscript using “track changes” (as required by the journal). We also submitted a copy of the manuscript accepting all of these changes. Reviewers’ comments were copied and pasted here. All comments were put in quotes, italicized and marked with the blue color for ease of reading. All page numbers here refer to the manuscript with markup or “track changes”.

Sincerely,

Maria Wong, Ph.D.

Professor, Idaho State University and Corresponding Author

**Editor**

*“Journal Requirements:*

*When submitting your revision, we need you to address these additional requirements.*

*1. Please ensure that your manuscript meets PLOS ONE's style requirements, including those for file naming. The PLOS ONE style templates can be found at*

[*https://journals.plos.org/plosone/s/file?id=wjVg/PLOSOne_formatting_sample_main_body.pdf*](https://journals.plos.org/plosone/s/file?id=wjVg/PLOSOne_formatting_sample_main_body.pdf) *and*

[*https://journals.plos.org/plosone/s/file?id=ba62/PLOSOne_formatting_sample_title_authors_affiliations.pdf*](https://journals.plos.org/plosone/s/file?id=ba62/PLOSOne_formatting_sample_title_authors_affiliations.pdf)*”*

We reviewed all the formatting requirements of the journal in the two templates. Changes were made to meet PLOS ONE’s style requirements. If we misunderstood or missed some requirements, please let us know and we will make the changes immediately.

*“2. Please include your full ethics statement in the ‘Methods’ section of your manuscript file. In your statement, please include the full name of the IRB or ethics committee who approved or waived your study, as well as whether or not you obtained informed written or verbal consent. If consent was waived for your study, please include this information in your statement as well.”*

This information is included in the revised manuscript.

“This study was approved by Emory University Institutional Review Board (exempt), Mercer University Institutional Review Board and Idaho State University Human Subjects Committee,

University We obtained informed consent from all participants.” (p.8)

**Reviewer 1**

*3. “There is a large zip file at the end of the article that I was unable to access or open. Some of the questions I have might be answered there. But the fact remains that the file couldn't be opened after quite a long time of trying.”*

The journal requires supplementary data files to be zipped. I will consult with the editorial staff about zip files. If that is not a requirement, I will not zip any files. In the original submission, I included a SPSS data file. In case reviewers do not use SPSS, this time I also included a tab-delimited file containing the same data that we used to do the analyses for this paper.  *4. “First point: "In other words, after controlling for other variables in the analyses, participants who admire their favorite celebrity almost exclusively because of their ability to entertain, were significantly less likely to engage in obnoxious celebrity stalking." This point has been made in other publications, the fact that being a fan and all of this other negative behavior are not related at all. In fact, in this case, being a "normal" fan makes it LESS likely that the person would be a stalker. Given the ways in which average fans are stigmatized in our culture, it seems important that this statement not be buried at the end of the article. It should be stated in the abstract, as it is an important finding.”*

The reviewer made a great point about the need to differentiate between “fan” and celebrity stalker” (also see our response below). We clarified the findings in the Discussion section.

“It is worth noting that the zero-order correlation between CAS Entertainment-social and our celebrity stalking measure was somewhat lower (though in the same direction) than the correlations between CAS Intense-personal and Borderline pathological and our celebrity stalking measure. In the multiple regression analyses, the relationship between CAS entertainment-social is opposite in direction from the other predictors of obnoxious celebrity stalking. In other words, after controlling for other variables in the analyses, participants who admired their favorite celebrity almost exclusively because of their ability to entertain, were significantly *less* likely to engage in obnoxious celebrity stalking. This finding is consistent with a body of research and the absorption-addiction model showing that individuals who admire their favorite celebrity only for entertainment and social reasons are likely to be more mentally healthy than those who become addicted [1-3]. In contrast, both CAS intense-personal and CAS borderline-pathological positively predict OFAS-18. Those who have personal thoughts about their favorite celerity frequently and feel compelled to learn more about them are more likely to engage in celebrity stalking.” (p. 20-21)

Per the reviewer’s suggestion, we summarized these findings in the Abstract (italicized).

“The stalking of celebrities is a serious issue for thousands of celebrities worldwide who are occasionally confronted by fans who merit the label “fanatic.” We administered measures of obnoxious celebrity stalking, celebrity worship, persistent pursuit of celebrities, threat directed toward celebrities, boredom susceptibility, disinhibition, experience seeking, thrill and adventure seeking, relationship styles, and anger to 596 college students from the U.S.A. We developed a model consisting of all but the latter five measures that successfully predicted actual obnoxious stalking behaviors of celebrities. Our results partially replicate earlier research and present some new findings. *Individuals who have personal thoughts about their favorite celerity frequently, feel compelled to learn more about them, pursue them consistently, threatened to harm them and were prone to boredom were more likely to engage in celebrity stalking. Controlling for these predictors, individuals who admire their favorite celebrity almost exclusively because of their ability to entertain were less likely to engage in celebrity stalking.*” (p.2)

*5. “The 18 items on the "obnoxious fan behavior" scale should be included in the article as they are focal and centrai to the findings. Have the authors validated this scale by submitting it to a panel of celebrities and seeing what percentage of them agree with the items? Defining a behavior as obnoxious in the absence of such a procedure seems questionable at best. For example, if you asked a celebrity if they found a "fan" expressing the opinion that they are attractive as obnoxious, how many of them would agree with that? In my own experience and observations, a large percentage of celebrities would simply be flattered. Also the situation under which this might happen would make a huge difference in how it might be received.”*

This scale was validated in a study done in 2016 [4]. We include all items of OFAS-18 in the supplementary material and mentioned in the text that we did so.  *6. "Further research should be conducted on a sample of more serious celebrity stalkers, a sample that presumably would score much higher on our OFAS-18 measure." This statement concerns me. If the current instrument is an early attempt at a way to identify celebrity stalkers, then how would a sample of "more serious celebrity stalkers" be obtained? The reasoning here feels somewhat circular to me.”*

We modified this statement in the last paragraph of the Discussion section to clarify our idea and reasoning.

“Our study is not without some limitations. As with any correlational research, it is impossible to determine causation. Furthermore, our research relied on self-report data. Also noteworthy is the fact that we cannot generalize our findings to the general population. Table 1 shows that the vast majority of our sample scored rather low on the OFAS-18, our measure of celebrity stalking behavior. It remains to be seen whether research conducted on a sample of more serious celebrity stalkers will yield findings similar to the one reported in this study. Another limitation is that our data were collected from Idaho and Georgia. Perhaps data collected from Hollywood would yield higher scores on OFAS-18.” (p.22) *7. “Overall this is a good study, with solid data and analysis. I like it. However, more care needs to be taken to make clear that the construct "fan" and the construct "celebrity stalker" are completely different and unrelated. The stalkers identified in the article (Chapman, Hinkley) were not "fans" in the truest sense of the word and many others are not as well (although certainly a few might be). An attempt to find stalkers among "fans" seems doomed to failure. And the idea that this could happen contributes to the stigma of being a typical media fan.”*

Thank you for the compliment. As we stated above, the reviewer made a great point about the need to differentiate between “fan” and celebrity stalker”. We address this issue in the both the Introduction and Discussion.

“There is evidence linking scores on the CAS with a measure of condoning celebrity stalking in others. That is, as admiration for one’s favorite celebrity increased, so did the tendency to condone the stalking of celebrities in others [4, 5]. There is also evidence that scores on the *Celebrity Attitude Scale* are significantly related to OFAS-18 scores [4]. It is important to note that these findings did not mean that fans and celebrity stalkers are the same. The moderate association between fans and celebrity stalkers indicate that even though there are individuals who are in both categories, there are also individuals who are fans but not celebrity stalkers and vice versa. It is therefore important to identify the factors that help us differentiate between these two constructs.” (p.4-5, Introduction)

“It is worth noting that the zero-order correlation between CAS Entertainment-social and our celebrity stalking measure was somewhat lower (though in the same direction) than the correlations between CAS Intense-personal and Borderline pathological and our celebrity stalking measure. In the multiple regression analyses, the relationship between CAS entertainment-social is opposite in direction from the other predictors of obnoxious celebrity stalking. In other words, after controlling for other variables in the analyses, participants who admire their favorite celebrity almost exclusively because of their ability to entertain, were significantly *less* likely to engage in obnoxious celebrity stalking. This finding is consistent with a body of research and the absorption-addiction model showing that persons who admire their favorite celebrity only for entertainment and social reasons are likely to be more mentally healthy than those who become addicted [1-3]. In contrast, both CAS intense-personal and CAS borderline-pathological positively predict OFAS-18. Those who have personal thoughts about their favorite celerity frequently and feel compelled to learn more about them are more likely to engage in celebrity stalking.” (p. 20-21, Discussion)

**Reviewer 2**

*8. “Thank you for the opportunity to review manuscript # PONE-D-22-09764, titled “Predicting the Stalking of Celebrities from Measures of Persistent Pursuit and Threat Directed toward Celebrities, Sensation Seeking and Celebrity Worship”. Using a large sample of undergraduate and graduate students across three university campuses, the present study examined associations between among a variety of self-report scales, including obnoxious celebrity stalking, celebrity worship, persistent pursuit of celebrities, threat directed toward celebrities, boredom susceptibility, disinhibition, experience seeking, thrill and adventure seeking, relationship styles, and anger. Zero-order correlations and multiple linear regression were used to conduct inferential analyses. I hope the author finds the following comments and questions helpful.”*

We appreciate the detailed feedback of Reviewer 2. In the space below, we provided a response to each comment and suggestion. When changes were not made, we provided an explanation. All participants were undergraduate students.  *9. “Note that Cohen’s d is transformed to Pearson’s correlation coefficient using the following equation: r = d/sqrt(d^2+4) (written in R code). So, d = .15 transforms to r = .07478995 and R2 = .005593536. The author reports that d = .15 “corresponds to an R2 of approximately .13” and cite Cohen (1988), but this is incorrect, as shown above. Infamously, despite his genius, Cohen (1988) got the transformations between common effect sizes wrong. Please correct the reported R2 and omit the citation to Cohen (1988), as it may be the only time he got something wrong.”*

We deleted the Cohen reference and redid the power analyses using parameters that are more similar to our analyses. A description of the power analysis can be found on p.9.

“An a priori power analysis using G*Power (Faul et al., 2007) indicated that a total sample size of 123 would be needed to detect a medium effect size of *d* ≥ 0.15 with 11 predictors in a multiple regression model with 80% power and alpha at .05.” (p.9) *10. “As measurement error is bound to vary from study to study, I see no value in reporting “In a previous study Cronbach’s alpha was .95” (line 154).”*

Even though Cronbach’s alpha varies from study to study, we reported Cronbach’s alphas from a previous study and this study to show that OFAS-18 has consistently high reliability (items have high internal consistency) in both studies.

“The *Obnoxious Fan Activities Scale-18* (OFAS-18) is a reduction from the 60-item *Fan Activities Scale* [6], a list of behaviors that fans sometimes direct toward celebrities. This reduction was accomplished by eliminating ‘normal’ fan activities (e.g., seeking autographs, going to shows at which the celebrity is appearing), ambiguous items (writing to a celebrity may or may not be normal depending on the content), and reducing overlap (“expressing sexual interest” is similar to “sexually coercing her/him”). The remaining 18 items went far beyond the range of normal fan behaviors. Every item was prefaced by the following question: “Since the age of 16, how often, if at all, have you ever engaged in any of the following activities?” Answers ranged from 1, “never,” to 5, “frequently.” Sample items include “following the celebrity while out in public,” going to or waiting at the celebrity’s hotel,” “expressing attraction or sexual interest,” and “trespassing on the celebrity’s property.” High scores indicate actual celebrity stalking behavior. *In a previous study Cronbach’s alpha was .95 [4]. Cronbach’s alpha for the OFAS-18 in the current study was .91.*” (p.9-10)

*11. “Related, the author reports Cronbach's alpha for self-report scales, but many have argued that Cronbach’s alpha is not a valid measure of internal consistency (McDonald, 1999; Revelle & Zinbarg, 2009; Sijtsma, 2009; Zinbarg et al., 2005). What some may consider the “actual” reliability scores may be higher or lower when obtained using McDonald’s omega index. It can’t hurt to follow the cited authors and report alpha, omega total, and omega hierarchical. The omega indices can be easily calculated using the ‘psych’ package in R.”*

Even though the Cronbach’s alpha has its shortcoming, it also has many strengths. It is easy to interpret and widely used by researchers. We decided to keep this measure in the article.

*12. “More descriptive statistics should be reported for self-report scales, including skew and kurtosis.”*

We included the skewness and kurtosis statistics of all variables in the supplementary material. There is still no agreement among researchers about what an acceptable range of skewness and kurtosis statistics should be. The z-tests for the two statistics are very sensitive to slight deviation from normality. We decided to follow the suggestions of recent publications [7-9]. For a more detailed discussion on this issue, see Dr. Simon’s website,

<https://imaging.mrc-cbu.cam.ac.uk/statswiki/FAQ/Simon>)

These publications recommend using a moderate range of skewness (a range between -2 and +2 is considered normal) and kurtosis (a range between -7 and +7 is considered normal) values to evaluate whether variables are considered normal. All variables have skewness and kurtosis statistics that are within the range of these recommended values except the dependent variable, OFAS-18 (skewness=7.13(se=0.10), kurtosis=59.55(se=0.20)) and ORI & CS Threat (skewness=3.87(se=.10), kurtosis=18.84(se=0.20)). We decided not to transform either OFAS-18 or ORI & CS Threat, as any transformation (e.g., log transformation, inverse transformation) makes it difficult to interpret the relationship between a predictor and the dependent variable. It is useful to note that ORI & CS Threat is a significant predictor in the multiple regression model reported in the paper, as well as the Poisson regression model (*Wald χ^2^(1)*=22.09, *p*<.001) (see below). See our discussion on the distribution of OFAS-18 in #13.

*13. “Based on the mean (19.24) standard deviation (3.80) and possible range of scores (18-90), it is clear that the Obnoxious Fan Activities Scale was positively skewed and likely zero-inflated. This is problematic because it is the dependent variable in multiple linear regression, which almost guarantees that the residual errors are not normally distributed, and ordinary least squares regression is highly sensitive to deviation from normality. Thus, the regression coefficients, standard errors, and p-values from the multiple linear regression cannot be trusted. The author should think carefully about how to deal with the positive skew of the dependent variable. Many different types of robust regression methods have been developed for this purpose.”*

See our response to #12. The reviewer is right about the positive skewness of OFAS-18. About 64% of participants had a score of 18, which means they never engaged in any behaviors on the scale. After careful consideration, we analyzed the data using Poisson regression, a generalized linear model that takes into account the positive skewness of the dependent variable.[10, 11] Though OFAS-18 is not a count variable, Poisson regression takes into account the positive skewness of the variable. The results show that the CAS Intense Personal (*Wald χ^2^(1)*=8.61, *p*=.003) , CAS Borderline pathological (*Wald χ^2^(1)*=3.97, *p*=.046) and ORI & CS Threat are significant predictors (*Wald χ^2^(1)*=22.09, *p*=.000). CAS Entertainment Social is marginally significant (*Wald χ^2^(1)*=3.27, *p*=.07). These predictors are all significant in the multiple regression models. ORI & CS Persistent Pursuit (*Wald χ^2^(1)*=2.52, *p*=.11) and BSSS Boredom Susceptibility (*Wald χ^2^(1)*=2.46, *p*=.12) has a p-value of about .1. Though not statistically significant in the Poisson regression model, these p-values are lower than all of the remaining predictors. Thus, the results of the Poisson regression model are largely consistent with the analyses presented in the paper. We did not analyze the data using zero-inflated Poisson regression because doing so changes the nature of the questions addressed in this paper.

*14. “On lines 153-153, the author writes “High scores indicate actual celebrity stalking behavior”, referring the Obnoxious Fan Activities Scale. However, because data were collected exclusively from students living in Idaho and Georgia, I doubt seriously that many opportunities were presented to the study participants to follow a celebrity while out in public, go to or wait at a celebrity’s hotel, or trespass on celebrity’s property. Given that such stalking behavior is the focal study variable of interest, did the author sample from the appropriate geographic regions? I would be more convinced in the veracity of student’s self-reports on this scale if data had been collected in LA or NYC.”*
The reviewer makes a good point. Perhaps OFAS-18 scores would have been higher if we had sampled from Hollywood. We mentioned this in the study limitations in the Discussion section.

*15. “Related, were participants asked to report the name of the celebrity they claimed to stalk? How much actual celebrity stalking was measured in the current study? Again, based on the few descriptive statistics that were reported, it appears very little.”*

Also see response to #13. The participants were not asked to report the name of the celebrity they claimed to stalk. The range of average OFAS scores is 19 to 59. Participants reported engaging in all 18 types of stalking behaviors sometimes, i.e., “seldom” (a score of 2) and “occasionally” (a score of 3). Participants reported engaging in 11 out of 18 types of stalking behaviors more frequently, i.e., “often” (a score of 4) and “frequently” (a score of 5). For more information, please refer to the OFAS-18 measure and the data file in the supplementary material.

*16. “Regarding the ORI & CS, the author writes “factor analysis identified two factors” without reporting any information on the factor analysis that was allegedly performed. This is very strange. More details need to be reported on the factor analysis that was performed. What factor extraction techniques were utilized? Sequential model tests, the Hull Method, the Empirical Kaiser Criterion, traditional and revised parallel analysis? What estimator was used? What type of factor rotation was used? Factor loadings should be reported. What was the correlation between the two factors, that is, assuming an oblique rotation. Please report on all of the above.”*

Please refer to the paper that validated the ORI & CS scale [5].  *17. “Again, I’m open to be convinced otherwise but I see absolutely no value in reporting Cronbach’s alpha from another study. The author does this for all self-report scales.”*

See our response to # 10 and 11.  *18. “The author’s report that “Cronbach’s alpha in the current study for persistent pursuit was .97 and .98 for threat”, which is troubling. Such high Cronbach’s alphas usually indicate redundancy in item content and/or failure of a measure to capture the full breadth of content space, which is antithetical to high internal validity.”*

Please refer to the paper that validated the ORI & CS scale [5]. *19. “What does the author mean by, “good psychometric properties” (line 166)? Please elaborate.”*

On lines 181-185 we cited several studies that demonstrated specific examples of high reliability and validity of the CAS, and cited a review that offered additional evidence*.

20. “The author makes eye-ball average comparisons between the scales in the current study with previous studies (lines 221-230), which is bizarre. The whole point of inferential statistics is to obviate this type of imprecise, wishy-washy, eye-ball comparison. Has the author never heard of an independent samples t-test? This could be calculated without access to the raw data from previous studies. Technically, given that many of the variables were not normally distributed, the author should use a Mann-Whitney U test to determine if the distributions of scores varied across studies, although this would require access to the previously published data.”*

We are aware of the statistical tests mentioned in the comment. However, we would like to describe the findings in a more qualitative way in the beginning of the Results section. *21. “The author should consider making better use of the upper off-diagonal cells of Table 2. The author could report either non-parametric correlations (e.g., Kendall’s tau) or partial correlations controlling for demographic variables (age, gender, and race/ethnicity).”*

We believe that Tables 2 and 3 provide enough information for readers to understand the relationships among variables. All regression analyses statistically controlled for sex, age and the white race (0=no, 1=yes). These demographic variables were not significant in any regression models. They were dropped from the final model presented in the paper.  *22. “It would be good to report 95% confidence intervals for standardized regression coefficients in Table 3.”*

We reported the 95% confidence intervals (CI) of unstandardized regression coefficients in Table 3, as this determines the statistical significance of the coefficients. If the CI does not include 0, the coefficient is significant at *p*<.05.

*23. “It would also be good to report exact p-values in Table 3, instead of p-value cut-offs using asterisks.”*

Exact p-values are now reported in Table 3.

*24. “Note, most researchers no longer favor a backward elimination procedure because all of the automated, sequential, model comparisons have a high likelihood of capitalizing on sampling variability, increasing the likelihood of type-I error.”*

We eliminated this from the results section. We discussed our findings of stepwise procedure in a more general way.

“Multiple linear regression using these measures to predict OFAS-18. Sex, age and race (0=non-white and 1=white) were controlled for in all analyses. These demographic variables were not significant in any model. They were therefore from the final models presented here. The results showed that the three subscales of CAS, the two subscales of ORI & CS and BSSS Boredom susceptibility uniquely and significantly predicted OFAS-18. The model significantly explained about 22.4% of the variance of OFAS-18, *F*(11,587) =15.42, *p*<.001, *R^2^*=.224. The unstandardized and standardized beta coefficients, the *t* and *p* values were presented in Table 3. We entered the variables simultaneously for this analysis. We also conducted several stepwise multiple regression models and found that the same six variables significantly predicted scores on OFAS-18.” (p.16-17)

*25. “There was considerably heterogeneity in terms of demographic characteristics in the current study. Age, sex, and race/ethnicity should be included in the multiple linear regression as covariates to ensure the documented associations are not confounded by these variables. This is generally standard practice in studies with non-experimental correlational designs.”*

All regression analyses statistically controlled for sex, age and the white race (0=no, 1=yes). These demographic variables were not significant in any regression models. They were dropped from the final model presented in the paper. *26. “There is a clustered pattern to the observations in the present study. Schools were nested within different states (Idaho and Georgia), classrooms were presumably nested within different schools, and participants were nested within classrooms. This clustered or nested structure introduces potential non-independence among residuals, which is another violation of an underlying assumption of multiple linear regression, which renders the results untrustworthy. The author either needs to conduct multi-level modeling or specify a cluster variable and implement a sandwich estimator to account for the non-independence of observations, otherwise the standard errors of regression coefficients will be biased.”*

There are no statistically differences on the major variables across the three campuses. So we do not think that it is necessary to use multi-level models to analyze the data.  *27. “The interpretation of results is problematic. For example, the author writes “The fact that mean scores we obtained on our study variables were consistent with mean scores found in previous studies suggests that our participants showed diligence and conscientiousness in responding to our scales. This, combined with high Cronbach alphas, allows us to place considerable faith in our results.” First, as previously noted, many have argued that Cronbach’s alpha is not a valid measure of internal consistency. Moreover, the above interpretation would only sound if (1) participants showed diligence and conscientiousness in responding in previous studies, for which no evidence was provided, (2) there were neither measured nor unmeasured confounders that differed across studies, and (3) the samples in previous studies were drawn from the same population as the samples in the current study. Consequently, the quoted interpretation is tenuous at best.”*

We changed the wording in lines 282-285 to reflect the fact that there was consistency over several studies using the CAS, the ORI & CS, and OFAS-18 in mean scores and alphas, giving us confidence that the scores we obtained were valid.

“The main objective of this study was to assess the association between OFAS-18, a celebrity stalking measure, with measures of celebrity worship, persistent pursuit of celebrities, threat directed toward celebrities, boredom susceptibility, disinhibition, experience seeking, thrill and adventure seeking and relationship styles. The mean scores we obtained on our study variables were consistent with mean scores found in previous studies using the CAS, OFAS-18 and the ORI & CS. The consistency over time and over many studies combined with high Cronbach alphas, allows us to place considerable faith in our results. We should note, however, that adding one item to each of the four subscales of the BSSS did little to raise its internal reliability value.” (p.19)  *28. “Other sections of the discussion are equally problematic. For example, the author writes “Our measure of attachment correlated weakly (-.16) with ORI & CS in one of the 2006 studies (McCutcheon, Scott, Jr. 2006) in part because of a large sample size (n = 299).” However, a large sample size is not an explanation for a weak correlation. A large sample size is an explanation for why a weak correlation would be statistically significant at p < .05, but it’s not an explanation for the small point estimate (i.e., not an explanation for the weak correlation itself).”*

We modified our statement to address the reviewer’s concern (changes were marked by the underlined text).

“Why did anger and attachment not predict OFAS-18 scores? Previous research linking anger with OFAS-18 scores was only indirect and weak. That is, anger correlated weakly with a measure of stalking in general in one of the 2006 studies [5], but was not included as a variable in the other celebrity stalking study from the same year [12], nor was it included in the 2016 study [4]. Our measure of attachment correlated weakly but significantly (-.16) with ORI & CS in one of the 2006 studies (McCutcheon, Scott, Jr. 2006) in part because of a large sample size (*n* = 299).” (p.19)

*29. “In regard to “Why did anger and attachment not predict OFAS-18 scores?” (line 268). Did the author considered multicollinearity among independent variables and consequent inflation of standard errors? This could be probed empirically by calculating variance inflation factors for the predictors in the multiple regression.”*

We agree that multicollinearity is an important issue in multiple regression. We examined the variance inflation factors (VIF) among the predictors. Generally, a good rule-of-thumb is that a VIF greater than 4 indicate that the predictors should be examined while a VIF that exceeds 10 indicate serious multicollinearity (<https://online.stat.psu.edu/stat462/node/180/>). A VIF of 1 means that there is no correlation among a predictor and other predictors. None of our predictors have a VIF that exceeds 4. The VIF of all predictors ranges from 1.06 to 2.97. This indicates that there is no multicollinearity in the predictors.  *30. “The limitations that the author noted in the last paragraph of the discussion, although correct, further dampen my enthusiasm for the current study. Mainly, the present study was a cross-sectional, non-experimental design that relied solely on self-report scales that were completed by psychology students, which weakens both internal and external validity.”*

Many published studies use cross-sectional data and a non-experimental (correlational) design. Even though some questions should be answered using a longitudinal design, cross-sectional data also allow researchers to address important questions and save time and resources. We believe that our research questions are adequately addressed by a cross-sectional design with correlational data. Some variables cannot be ethically studied in a research lab. Manipulating stalking and celebrity worship in a laboratory setting is not ethical and difficult to accomplish. Given the nature of our research questions, we believe that a cross-sectional design using correlational data is sufficient. Even though we cannot ascertain causal relations among OFAS-18 and the predictors, we found significant and meaningful associations that inform future research. We believe that our choice of research design does not affect the internal validity (whether the design, procedure and analysis addressed the research questions) and external validity (generalizability) of the findings.

*31. “I don't believe the present study warrants publication in PLOS ONE. Nevertheless, I hope the above comments and questions are helpful. Best wishes and good luck.”*

Thank you for the feedback. We attempted to address the reviewer’s concerns in the response letter and in the revised manuscript.  *“References:

McDonald, R. P. (1999). Test theory: A unified treatment. Test theory a unified treatment. Hillsdale, NJ: Lawrence Erlbaum. doi:10.4324/9781410601087

Revelle, W., & Zinbarg, R. E. (2009). Coefficients alpha, beta, omega, and the glb: Comments on Sijtsma. Psychometrika, 74(1), 145-154.

Sijtsma, K. (2009). On the use, the misuse, and the very limited usefulness of Cronbach's Alpha. Psychometrika, 74(1), 107–120. doi:10.1007/s11336-008-9101-0

Zinbarg, R. E., Revelle, W., Yovel, I., & Li, W. (2005). Cronbach’s α, Revelle’s β, and McDonald’s ω H: Their relations with each other and two alternative conceptualizations of reliability. Psychometrika, 70(1), 123-133.”*

These are references provided by Reviewer 2. The reference we used in this letter are listed below.

**References**

1.	Maltby J, McCutcheon LE, Lowinger RJ. Brief report: Celebrity worshipers and the five-factor model of personality. North American Journal of Psychology. 2011;13(2):343-8. PubMed PMID: 2011-17052-012.

2.	Maltby J, Houran J, McCutcheon LE. A clinical interpretation of attitudes and behaviors associated with celebrity worship. Journal of Nervous and Mental Disease. 2003;191(1):25-9. doi: 10.1097/00005053-200301000-00005. PubMed PMID: 2003-04332-007.

3.	Maltby J, Day L, McCutcheon LE, Houran J, Ashe D. Extreme celebrity worship, fantasy proneness and dissociation: Developing the measurement and understanding of celebrity worship within a clinical personality context. Personality and Individual Differences. 2006;40(2):273-83. doi: 10.1016/j.paid.2005.07.004. PubMed PMID: 2006-01509-010.

4.	McCutcheon LE, Aruguete M, McCarley NG, Jenkins WJ. Further validation of an indirect measure of celebrity stalking. Journal of Studies in Social Sciences. 2016;14(1):75-91.

5.	McCutcheon LE, Aruguete M, Scott VB, Jr., Parker JS, Calicchia J. The Development and Validation of an Indirect Measure of Celebrity Stalking. North American Journal of Psychology. 2006;8(3):503-16. PubMed PMID: 2006-22967-011.

6.	Spitzberg BH, Cupach WR. Fanning the flames of fandom: Celebrity worship, parasocial interaction, and stalking. In: Meloy JR, Sheridan L, Hoffmann J, editors. Stalking, threatening, and attacking public figures: A psychological and behavioral analysis. New York, NY: Oxford University Press; 2008. p. 287-321.

7.	Byrne BM. Structural equation modeling with AMOS: Basic concepts, applications, and programming, 2nd ed. New York, NY: Routledge/Taylor & Francis Group; 2010.

8.	Hair J, Black WC, Babin BJ, Anderson RE. Multivariate data analysis. 7th ed. Upper Saddle River, New Jersey: Pearson Educational International; 2010.

9.	Curran PJ, West SG, Finch JF. The robustness of test statistics to nonnormality and specification error in confirmatory factor analysis. Psychological Methods. 1996;1(1):16-29. doi: 10.1037/1082-989X.1.1.16. PubMed PMID: 1996-03170-002.

10.	Atkins DC, Baldwin SA, Zheng C, Gallop RJ, Neighbors C. A tutorial on count regression and zero-altered count models for longitudinal substance use data. Psychology of Addictive Behaviors. 2013;27(1):166-77. doi: 10.1037/a0029508

10.1037/a0029508.supp (Supplemental). PubMed PMID: 2012-22398-001.

11.	Atkins DC, Gallop RJ. Rethinking how family researchers model infrequent outcomes: A tutorial on count regression and zero-inflated models. Journal of Family Psychology. 2007;21(4):726-35. doi: 10.1037/0893-3200.21.4.726

10.1037/0893-3200.21.4.726.supp (Supplemental). PubMed PMID: 2007-18728-018.

12.	McCutcheon LE, Scott VB, Jr., Aruguete MS, Parker J. Exploring the Link between Attachment and the Inclination to Obsess about or Stalk Celebrities. North American Journal of Psychology. 2006;8(2):289-300. PubMed PMID: 2006-09074-009.
