# Supplementary material for: Predicting the stalking of celebrities from measures of persistent pursuit and threat directed toward celebrities, sensation seeking and celebrity worship
Source: PLoS One. 2023 Mar 1;18(3):e0281551. doi: 10.1371/journal.pone.0281551 (PMC9977013; doi:10.1371/journal.pone.0281551)
Supplement: Attachment — Submitted filename: response to editors & reviewers plos 1-4.docx [file pone.0281551.s004.docx]

January 4, 2023

Dear Dr. Rowe:

Thank you for the opportunity to submit a second revision of the manuscript. We responded to each comment/suggestion in the space below. Whenever possible, changes were made in the revised manuscript per the editor and reviewers’ suggestions. When changes were not made, we provided a detailed explanation in the response letter. Changes were noted in the revised manuscript using “track changes” (as required by the journal). We also submitted a copy of the manuscript accepting all of these changes. Reviewers’ comments were copied and pasted here. All comments were put in quotes, italicized and marked with the blue color for ease of reading. All page numbers here refer to the manuscript with markup or “track changes”.

Sincerely,

Maria Wong, Ph.D.

Professor, Idaho State University and Corresponding Author

*1. “In relation to Reviewer 2's comments:*

*Point 13. Please include details of the Poisson regression modelling in your manuscript, including the rationale for conducting it and the extent to which the findings are similar to your current modelling.”*

We included this information on p.19.

“The dependent variable OFAS-18 is positively skewed. About 64% of participants reported that they did not engage in any celebrity stalking behaviors and most participants had a lower score. We therefore supplemented the main analyses by conducting Poisson regression, a type of generalized linear models designed for skewed event data.[1, 2] The results showed that the CAS Intense Personal (*Wald χ^2^(1)*=8.61, *p*=.003) , CAS Borderline pathological (*Wald χ^2^(1)*=3.97, *p*=.046) and ORI & CS Threat were significant predictors (*Wald χ^2^(1)*=22.09, *p*=.000). CAS Entertainment Social was marginally significant (*Wald χ^2^(1)*=3.27, *p*=.07). These predictors are all significant in the multiple regression models. ORI & CS Persistent Pursuit (*Wald χ^2^(1)*=2.52, *p*=.11) and BSSS Boredom Susceptibility (*Wald χ^2^(1)*=2.46, *p*=.12) has a p-value of about .1. Though not statistically significant in the Poisson regression model, their p-values were lower than all of the remaining predictors. Thus, the results of the Poisson regression model are largely consistent with the analyses presented in the paper.”

*2.“Point 16. Please add a few more details on the factor structure identified in the cited paper.”*

We provided additional information in the manuscript on p.5.

“Attempts to develop and validate an indirect measure of celebrity stalking resulted in the *Obsessive Relational Intrusion and Celebrity Stalking* scale (ORI & CS) [3]. Each of the 11 items presents a brief scenario in which the behavior of a fan is described and the respondent rates the inappropriateness of that behavior. Initial exploratory factor analysis revealed a two-factor solution [4], which was subsequently supported by a confirmatory factor analysis using additional data [3]. Seven items loaded on a factor labeled “persistent pursuit,” because of items like one in which a fan is depicted following a celebrity on several occasions. The “threat” factor was so named because it contained items that could potentially harm the celebrity, like one in which a fan writes a letter to a celebrity describing sexual acts that the fan would like to perform on the celebrity. As noted above, ORI & CS scores correlated significantly with OFAS-18 scores in a previous study [5].”

*3.“Point 22. I agree with the reviewer that the CIs on the standardised betas will be most helpful. The CIs there are as informative regarding significance as the CIs on the b coefficients.”*

We obtained the CIs of the standardized betas and presented them in Table 3.

*4.“Point 26. The reviewer's point is about correlations between observations from the same school; non-independent observations will reduce artificially reduce the variance in the Sample. This problem exists independently from mean level differences between schools. I think the reviewer’s suggestion of a clustered regression is a good one. So I would be grateful if you could run these additional analyses which I think are commonly available in most stats packages.”*

We ran a multi-level regression model with no predictors to estimate the amount of variance in OFAS-18 that is accounted for by the individuals and the schools (clusters) that they belong to.[6] The level-1 residual variance was statistically significant, 13.92 (se=.79), *p*<.001 while the level-2 residual variance component was not, .39 (se=.46), *p*=.39. This indicated that schools (clusters) did not significantly explain variance in OFAS-18, meaning that multi-level regression analyses were not warranted. We used these estimates to compute the intraclass correlation (ICC). The ICC was estimated to be .39/ (13.92+.39) = .02, meaning that roughly 2% of the variability in the OFAS-18 was attributed to schools. An ICC value of .05 or higher is typically used as the cut-off value to determine whether multi-level analyses are necessary.[6] Our data show that there is no significant clustering effect attributable to schools. The single-level regression analyses presented in the paper are therefore appropriate.

*5.“28. I agree with the reviewer that a small correlation does not result from a large sample size. Instead a large sample size provides the power to detect smaller correlations. So the text needs to be re-written to say that there may only be a small correlation between these measures in the population and therefore a large sample is required to identify it as signficant.”*

We agreed with the editor on this point. The original text mentioned issues of sample size and statistical significance. Upon careful consideration, we determined that it did not succinctly communicate our ideas. We rewrote the text to better convey our ideas.

“Anger and attachment did not predict OFAS-18 scores. Previous research linking anger with OFAS-18 scores was only indirect and weak. That is, anger correlated weakly with a measure of stalking in general in one of the 2006 studies [3], but was not included as a variable in the other celebrity stalking study from the same year [4], nor was it included in the 2016 study [5]. Our measure of attachment correlated weakly, though significantly (*r*=-.16) with ORI & CS in one of the 2006 studies [4]. The current study found non-significant associations between celebrity stalking (as measured by OFAS-18) and both anger and attachment. This is the first time that these relations were reported in the literature. Previous studies did not use OFAS-18 to measure celebrity stalking. Taken as a whole, both current and past findings indicate that celebrity stalking is either uncorrelated or weakly correlated with anger or attachment.” (p.21)

*6.“In my own reading of the revised manuscript:*

*Line 27. Celebrity not “celerity"”*

We corrected this typo.

*7.“Line 39. My understanding is that Princess Diana died in a car crash chased by the press rather than stalkers, so I recommend deleting this example.”*

We deleted this example.

*8.“Line 62. Please remove mention of the statistical test used and just focus on the substantive result.”*

We removed information about the statistical test.

“One study found that OFAS-18 scores correlated significantly with a measure of attraction to one’s favorite celebrity and two related measures of attitudes condoning celebrity stalking, namely persistent pursuit and threat. In the same study, OFAS-18 scores significantly predicted scores on attitudes condoning the threatening type of celebrity stalking [5].” (p.4)

*9.“Line 132. Again concentrate on the substantive point that your predictors will independently correlate with stalking rather than mentioning multiple regression.”*

We made this change.

“To summarize, we used scores from several scales that correlated with measures of either the condoning of celebrity stalking or actual stalking behavior. We predicted that the combination of these scores would significantly predict OFAS-18 as a measure of stalking.” (p.8)

*10.“Line 240 Head this section “Descriptive Statistics””*

This section heading was modified per your suggestion. The section includes findings on means, standard deviations and zero-order correlations on all variables.

*11. “Line 241-250 Move this comparison to previous studies to the discussion.”*

This comparison was moved to the Discussion section on p.19-20.

“Discussion

The main objective of this study was to assess the association between OFAS-18, a celebrity stalking measure, with measures of celebrity worship, persistent pursuit of celebrities, threat directed toward celebrities, boredom susceptibility, disinhibition, experience seeking, thrill and adventure seeking and relationship styles. The mean scores we obtained on our study variables were consistent with mean scores found in previous studies using the CAS, OFAS-18 and the ORI & CS. For example, the total CAS score of 51.91 is similar to the total CAS mean score (57.74) obtained by McCutcheon et al. [7], and our mean scores for ORI & CS Persistent pursuit (15.40) and ORI & CS Threat (5.93) are consistent with the mean scores (17.97 & 6.20 respectively) obtained by McCutcheon, Aruguete et al. [5], and the mean scores (17.55 & 4.75 respectively) obtained by McCutcheon et al. [3]. Our mean scores are not directly comparable to those obtained by Hoyle et al. [8] and Lopez-Bonilla and Lopez-Bonilla (2010) on the BSSS because they used the eight-item version, but both of their studies found that mean scores were highest on the Experience Seeking subscale, just as our study did. The consistency over time and over many studies, combined with high Cronbach alphas, allows us to place considerable faith in our results. We should note, however, that adding one item to each of the four subscales of the BSSS did little to raise its internal reliability value.” (p.19-20)

*12. Line 253 Head this section “Predictors of stalking” or something similar that conveys the contents of the section more clearly.*

As mentioned above, the first section of the Results section has a heading of “Descriptive statistics” (p.14). This section includes findings on means, standard deviations and zero-order correlations on all variables. We labeled the second section “ Predictors of celebrity stalking”. The section includes findings on multiple regression and Poisson regression (p.16).

*13. “Line 265. Clarify that the control of sex etc was in preliminary models.”*

We made this clarification on p.17.

“Multiple linear regression using these measures to predict OFAS-18. Sex, age and race (0=non-white and 1=white) were controlled for in all analyses. These demographic variables were not significant any model. They were removed from the final models presented here.” (p.17)

*14. “Line 267 insert “removed””*

We inserted this word per your suggestion.

**References**

1.	Atkins DC, Baldwin SA, Zheng C, Gallop RJ, Neighbors C. A tutorial on count regression and zero-altered count models for longitudinal substance use data. Psychology of Addictive Behaviors. 2013;27(1):166-77. doi: 10.1037/a0029508

10.1037/a0029508.supp (Supplemental). PubMed PMID: 2012-22398-001.

2.	Atkins DC, Gallop RJ. Rethinking how family researchers model infrequent outcomes: A tutorial on count regression and zero-inflated models. Journal of Family Psychology. 2007;21(4):726-35. doi: 10.1037/0893-3200.21.4.726

10.1037/0893-3200.21.4.726.supp (Supplemental). PubMed PMID: 2007-18728-018.

3.	McCutcheon LE, Aruguete M, Scott VB, Jr., Parker JS, Calicchia J. The Development and Validation of an Indirect Measure of Celebrity Stalking. North American Journal of Psychology. 2006;8(3):503-16. PubMed PMID: 2006-22967-011.

4.	McCutcheon LE, Scott VB, Jr., Aruguete MS, Parker J. Exploring the Link between Attachment and the Inclination to Obsess about or Stalk Celebrities. North American Journal of Psychology. 2006;8(2):289-300. PubMed PMID: 2006-09074-009.

5.	McCutcheon LE, Aruguete M, McCarley NG, Jenkins WJ. Further validation of an indirect measure of celebrity stalking. Journal of Studies in Social Sciences. 2016;14(1):75-91.

6.	Heck RH, Thomas SL, Tabata LN. Multilevel and longitudinal modeling with IBM SPSS: Routledge; 2013.

7.	McCutcheon LE, Green TD, Besharat MA, Edman JL, Wenger JL, Shabahang R. Values of college students in Iran and the United States who admire celebrities. Psychological Reports. 2021;124(1):299-317. doi: 10.1177/0033294119898119. PubMed PMID: 2021-07143-016.

8.	Hoyle RH, Stephenson MT, Palmgreen P, Pugzles Lorch E, Donohew RL. Reliability and validity of a brief measure of sensation seeking. Personality and Individual Differences. 2002;32(3):401-14. doi: 10.1016/S0191-8869(01)00032-0. PubMed PMID: 2002-00282-003.
