# Supplementary material for: Predicting the stalking of celebrities from measures of persistent pursuit and threat directed toward celebrities, sensation seeking and celebrity worship
Source: PLoS One. 2023 Mar 1;18(3):e0281551. doi: 10.1371/journal.pone.0281551 (PMC9977013; doi:10.1371/journal.pone.0281551)
Supplement: Attachment — Submitted filename: response to editors & reviewers plos 1-23.docx [file pone.0281551.s005.docx]

January 23, 2023

Dear Dr. Rowe:

Thank you for the opportunity to submit a third revision of the manuscript. Changes were noted in the revised manuscript using “track changes” (as required by the journal). We also submitted a copy of the manuscript accepting all of these changes. Reviewers’ comments were copied and pasted here. All comments were put in quotes, italicized and marked with the blue color for ease of reading. All page numbers here refer to the manuscript with markup or “track changes”.

Sincerely,

Maria Wong, Ph.D.

Professor, Idaho State University and Corresponding Author

*1. “Very many thanks for carefully addressing the points raised after the previous review. In my view, the paper is ready for publication after a some very minor amendments, as specified here:*

*You have dealt with the multi-level issue raised well. I would be grateful if you could include reference to your analysis testing the necessity for a multi-level approach somewhere in the manuscript. There is no need to provide the details, but please demonstrate you have considered the issue which may come to the minds of many readers.”*

Thank you very much for the positive feedback. We include a discussion of this issue in the manuscript.

We added the following paragraph to discuss whether it is necessary to analyze the data with multi-level regression models.

“The fact that participants came from three different universities raised the issue of whether multi-level regression was needed to analyze the data. To address this issue, we calculated the intra-class correlation (ICC) to determine the degree of variability in the dependent variable that could be attributed to schools (clusters). The ICC were estimated to be .02, meaning that roughly 2% of the variability in the OFAS-18 was attributed to schools. An ICC value of .05 or higher is typically used as the cut-off value to determine whether multi-level analyses are necessary.[1] Our data show that there is no significant clustering effect attributable to schools. The single-level regression analyses presented in the paper are therefore appropriate.” (p.18-19)

*2. “Line 245 are not is”*

This was changed.

“Mean scores for each scale are consistent with findings reported in previous studies.” (p.14)”

*3. “TAble 2. Abbreviations need to be defined in the Note”*

We added the definitions of all abbreviations in the Note of Table 2.

*4. “Line 272 insert “in””*

This was changed.

“These demographic variables were not significant in any model.” (p.16)

*5. “Line 290 delete unnecessary space.”*

The unnecessary space was deleted.

**References**

1.	Heck RH, Thomas SL, Tabata LN. Multilevel and longitudinal modeling with IBM SPSS: Routledge; 2013.
